# N-myc Downstream-Regulated Gene 2 (NDRG2) Function as a Positive Regulator of Apoptosis: A New Insight into NDRG2 as a Tumor Suppressor

**DOI:** 10.3390/cells10102649

**Published:** 2021-10-04

**Authors:** Gayeon Kim, Seyeon Lim, Kwang Dong Kim

**Affiliations:** 1 Division of Applied Life Science, Gyeongsang National University, Jinju 52828, Korea; kimga705@gnu.ac.kr (G.K.); yeon21@gnu.ac.kr (S.L.); 2 PMBBRC, Gyeongsang National University, Jinju 52828, Korea; 3 Division of Life Sciences, College of Natural Science, Gyeongsang National University, Jinju 52828, Korea

**Keywords:** N-myc downstream-regulated gene 2, tumor suppressor, apoptosis, molecular target for tumor therapy

## Abstract

N-myc downstream-regulated gene 2 (NDRG2) is a tumor suppressor gene that increases tumor sensitivity to anticancer drugs, slows tumor progression, and inhibits metastasis. NDRG2 is suppressed in various aggressive tumor positions, whereas NDRG2 expression is associated with patient prognosis, such as an improved survival rate. In this review, we summarize the tumor suppressor mechanism of NDRG2 and provide information on the function of NDRG2 concerning the susceptibility of cells to apoptosis. NDRG2 increases the susceptibility to apoptosis in various physiological environments of cells, such as development, hypoxia, nutrient deprivation, and cancer drug treatment. Although the molecular and cell biological mechanisms of NDRG2 have not been fully elucidated, we provide information on the mechanisms of NDRG2 in relation to apoptosis in various environments. This review can assist the design of research regarding NDRG2 function and suggests the potential of NDRG2 as a molecular target for cancer patients.

## 1. Introduction

The N-myc downstream-regulated gene (NDRG) family consists of NDRG1, NDRG2, NDRG3, and NDRG4. The proteins in this family are characterized by a sterase/lipase/thioesterase active site serine and an α/β hydrase fold of about 20 amino acids; however, they do not exhibit enzyme activity [1,2,3]. Among the family members, NDRG2 has been well studied regarding its structure and expression profiles in tissues [4]. As a tumor suppressor or stress response gene, NDRG2 is involved in the anti-metastasis and antiproliferation of tumor cells [5,6,7], and is implicated in responses to stress responses, which involve hormones, ions, body fluid metabolism [8,9,10], hypoxia, and lipotoxicity [11,12]. Notably, genome deletion or promoter methylation (or both) of the NDRG2 gene increases tumorigenesis by inhibiting NDRG2 expression in various tumors [13,14,15]. Furthermore, the expression of NDRG2 is closely related to tumor prognosis [6,16,17], which is associated with cancer metastasis inhibition and therapeutic outcome. The results of the treatment of tumors through radiation or drug therapy (or both) are closely related to the induction of apoptosis in tumor cells. In this review, we summarize the antitumor function of NDRG2 as a tumor suppressor gene and the function of NDRG2 in relation to apoptosis in various physiological environments as a prognostic tumor marker.

## 2. NDRG2 Functions as a Tumor Suppressor

Downregulated NDRG2 expression is closely related to poor prognoses, such as a reduction in overall survival and disease-free survival rates in tumor patients [6]. NDRG2 regulates the pathological processes associated with tumor aggressiveness, such as proliferation and invasion/epithelial–mesenchymal transition (EMT) in various tumors. NDRG2 regulates intracellular signals by inhibiting c-Jun phosphorylation and cyclin D expression, thus inhibiting cell proliferation [16]. NDRG2 overexpression was shown to decrease intracellular β-catenin levels and TCF/LEF activity by activating glycogen synthase kinase 3β (GSK-3β) in a colorectal carcinoma cell line, SW620. The inhibition of TCF/β-catenin activity by NDRG2 suppresses tumor metastasis [18]. The MMP (matrix metalloproteinase) family contributes to the degradation of the extracellular matrix in tumor progression and metastasis [19,20,21]. Furthermore, NDRG2 expression was shown to be associated with MMP downregulation in clear cell renal cell carcinoma (CCRCC) and hepatocellular carcinoma (HCC) [22,23]. Additionally, MMP expression is regulated through mechanisms such as ERK1/2 inhibition, NFκB signaling regulation, and TGFβ signaling inhibition by NDRG2 overexpression [17,22,24,25,26]. NDRG2 has a role as a PP2A recruiter, inhibiting NFκB signaling by inducing NFκB-inducing kinase (NIK) dephosphorylation [27]. *NDRG2* was shown to suppress the TGF-β1-mediated induction of MMP through the regulation of integrin α3 expression in hepatocarcinoma and integrin α6 expression in metastatic murine breast cancer cells (4T1), thereby suppressing the activation of latent extracellular TGF-β [17,26].

Various stimuli, such as the IL-6 family, EGF, and IGF, activate Janu kinase/signal transducer and activator of transcription (JAK/STAT) signaling [28]. Signal transducer and activator of transcription 3 (STAT3) plays a role in cell proliferation, survival, and invasion/metastasis as a tumorigenic player [29,30,31]. NDRG2 expression suppresses Snail expression at the transcriptional level and epithelial–mesenchymal transition (EMT) by inhibiting STAT3 [32]. Snail is a zinc-finger transcription regulator that inhibits E-cadherin expression and initiates EMT [33]. The silencing of suppressors of cytokine signaling (SOCS-1) contributes to the preferential activation of STAT3 by the JAK pathway [34]. The overexpression of NDRG2 in MBA-MB231 breast cancer cells increases SOCS-1 expression, and the JAK/STAT3 pathway is negatively regulated by SOCS-1 [35]. Although there are reports on the NDRG2-mediated regulation of signal transduction and EMT-inducing transcription factor, the exact molecular mechanism has not been fully elucidated.

**Figure 1:**
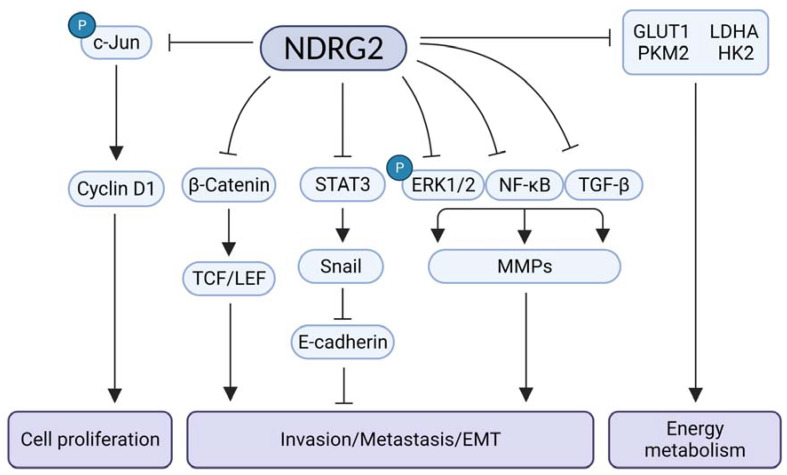
Overview of NDRG2-mediated antitumor activity. GLUT, glucose transporter; LDHA, lactate dehydrogenase A; PKM2, pyruvate kinase M2; HK2, hexokinase 2; TCF/LEF, T-cell factor/lymphoid enhancer factor; STAT3, signal transducer and activator of transcription 3; ERK1/2, extracellular signal-regulated protein kinase; NFκB, nuclear factor kappa-light-chain-enhancer of activated B cells; TGF-β, transforming Growth Factor-β; MMP, matrix metalloproteinase; EMT, epithelial-mesenchymal transition.

The Warburg effect indicates that cancer cells prefer metabolism through glycolysis over the much more efficient oxidative phosphorylation pathway that is favored by most other cells. Therefore, increased glucose consumption is required, as glucose is a carbon source for anabolic processes to support cell proliferation. An increase in glucose transporters (GLUTs) is required to enable large amounts of glucose to be taken up in tumors [36,37,38,39]. GLUT-1 promotes glucose transport across the plasma membrane of mammalian cells, and GLUT-1 overexpression is a prognostic biomarker of cancer [40,41] and can be a potential target, as it has been shown to regulate cancer in several reports [42,43,44]. NDRG2 expression was shown to inhibit glucose uptake by promoting the degradation of GLUT1 protein in breast cancer cells [8]. Catalytic enzymes, such as hexokinase 2 (HK2), pyruvate kinase M2 (PKM2), lactate dehydrogenase A (LDHA), and GLUT-1, are markedly inhibited by NDRG2 expression in colorectal cancer cells and patients [45]. Altogether, NDRG2 is an important prognostic marker that controls various cellular physiological pathways involved in tumor aggressiveness (Figure 1).

## 3. Proapoptotic Function of NDRG2

### 3.1. Spermatogenesis and NDRG2

Leydig cells are the main constituent of interstitial cells and produce testosterone for spermatogenesis in the testes [46]. Abnormalities of Leydig cells are prevalent in infertility diseases, and apoptosis of the cells was found in the testes of patients with maturation arrest and Sertoli cell-only syndrome (SCO) [47]. In Sprague–Dawley rats and the TM3 Leydig cell line treated with Leydig-cell-specific toxicant ethane dimethanesulfonate (EDS), NDRG2 is upregulated and translocated into the nucleus from the cytoplasm under apoptotic stimulation. The knockdown of NDRG2 induced increased proliferation and decreased apoptotic TM3 cells [48]. In the pathogenic process of cryptorchidism, p53 and proapoptotic proteins were upregulated in germ cells undergoing apoptosis, and p53 was associated with spontaneous germ cell death in the first wave of spermatogenesis [49,50]. NDRG2 and p53 expression was significantly upregulated in germ cells purified seven days after surgery, the upregulated expression of NDRG2 was associated with testicular germ cell apoptosis in cryptorchid testes [51]. Additionally, NDRG2 is a novel p53-inducible target involved in the p53-mediated apoptosis pathway [52]. Therefore, this suggests that NDRG2 is a novel gene involved in Leydig cell apoptosis and male fertility associated with p53 function. 

### 3.2. p53-Mediated Apoptosis and NDRG2

**Figure 2:**
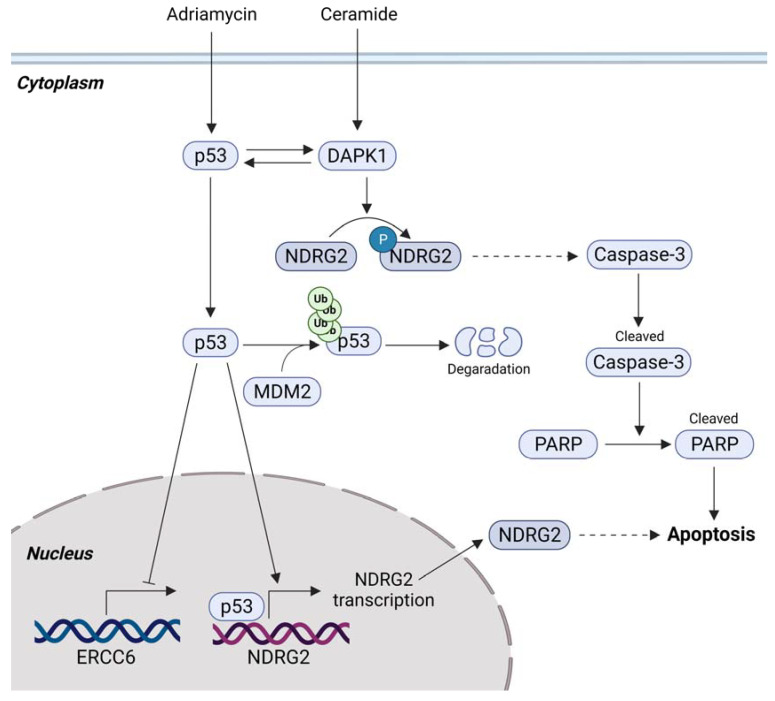
NDRG2 associated with p53-mediated apoptosis. NDRG2 is a novel p53-inducible target gene that is involved in p53-mediated apoptosis pathway. Unknown pathway (dotted arrow); DAPK1, death associated protein kinase 1; MDM2, mouse double minute 2; ERCC6, ERCC Repair 6; PARP, Poly (ADP-ribose) polymerase.

As a tumor suppressor, p53 is the most important factor that maintains genomic integrity [53], and it is widely accepted that p53-mediated apoptosis is essential for the tumor-suppressive activity of p53 [54,55]. NDRG2 is a new target gene that is regulated by p53. The level of mRNA and protein in NDRG2 is upregulated in a p53-dependent manner, and the silencing of NDRG2 attenuates p53-mediated apoptosis [52]. Furthermore, the overexpression of NDRG2 and p53 was shown to enhance the apoptosis of Huh7 cells (mutant p53) after Adriamycin-based chemotherapy and suppress the expression of the ERCC6 gene [56], which is involved in a sub-pathway of nucleotide excision repair and is associated with cancer drug resistance [57,58]. The expression of murine double minute gene 2 (MDM2) induces ubiquitination and mediates the degradation of wild-type p53, which promotes tumorigenesis [59]. The combination of MDM2 knockdown and NDRG2 overexpression inhibits cancer cell proliferation and induces apoptosis in vitro and in the xeno-transplantation model [60]. Additionally, NDRG2 is a substrate of novel death-associated protein kinase 1 (DAPK1), which promotes apoptosis induced by various stimuli and plays a role in tumor suppression [61,62,63,64]. The DAPK1-mediated phosphorylation of NDRG2 Ser350 promotes caspase-dependent apoptosis in neuronal cells treated with ceramide. DAPK1 increases p53 expression and p53 increases DAPK1 expression, suggesting a positive feedback regulation between DAPK1 and p53 [65,66]. DAPK1/p53/NDRG2 may play a role in apoptosis induced by various stimuli in several cell types (Figure 2). Altogether, NDRG2 plays a role in inducing p53-mediated apoptosis in tumor cells.

### 3.3. Sensitivity to Anticancer Drugs and NDRG2

**Figure 3:**
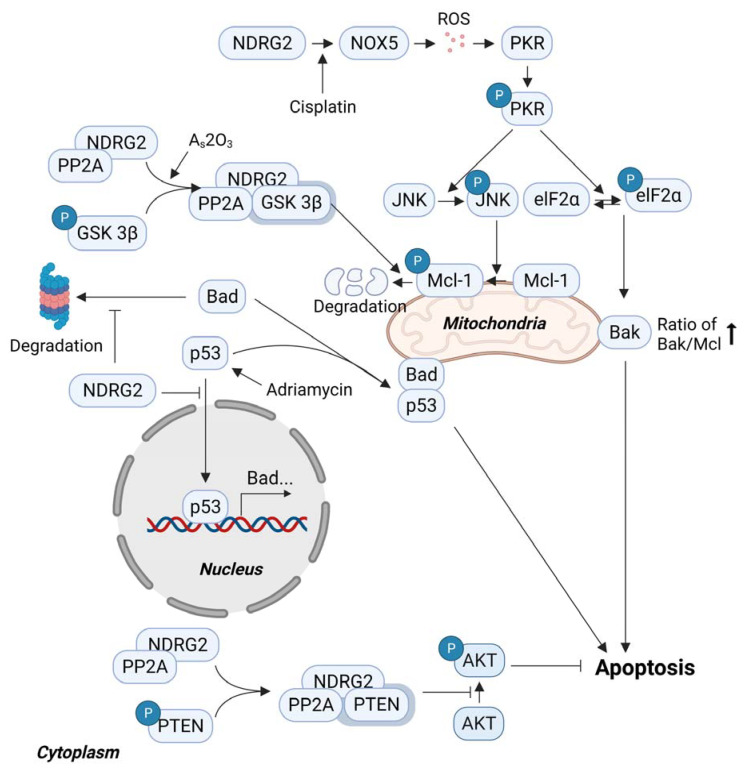
NDRG2 induces apoptosis to improve drug sensitivity in tumor cells. As an adaptor protein, NDRG2 increases the sensitivity of cells to apoptosis by mediating the PP2A-PTEN interaction and the PP2A-GSK3β interaction. NOX5 upregulation by NDRG2 enhances cisplatin-mediated apoptosis through ROS production. PP2A, protein phosphatase 2A; Mcl-1, myeloid leukemia cell differentiation protein-1; eIF, eukaryotic initiation factor; PKR, protein kinase R; NOX, NADPH oxidase; PTEN, phosphatase and tensin homolog).

The outcome of drug treatment for patients with cancer is an important factor that directly affects prognoses, such as survival and remission rates. There are several reports that show that NDRG2 contributes to drug sensitivity in tumor cells. NDRG2 overexpression enhanced the sensitivity of breast [67] and lung cancer cells [52] to Adriamycin in a p53-dependent manner. In the breast cancer cell line, NDRG2 overexpression prolonged the half-life of Bad and promoted the formation of the Bad/p53 complex in the mitochondria by inhibiting p53 from translocating into the nucleus [67]. NDRG2 also enhanced the sensitivity of an ovarian cancer cell line, SKOV-3, to pazopanib by activating the SK1/JNK1 signaling pathway [68]. NDRG2 enhanced the sensitivity to cisplatin and As_2_O_3_ in a p53 loss-of-function mutant myeloma cell line, U937 [69,70]. The degradation of Mcl-1 and the increase in Bak was mediated by JNK activation [71] and an increase in phospho-eIF2α, respectively, in NDRG2-overexpressed U937 cells after cisplatin treatment [69]. JNK activation and phospho-eIF2α were induced by PKR activation [72,73] through increased reactive oxygen species (ROS) mediated by NOX5 [74,75] induction in NDRG2-overexpressed U937. Furthermore, U937 cells were shown to be resistant to As_2_O_3_, a cancer drug used for myeloma [76]. NDRG2 overexpression induced Mcl-1 degradation and apoptosis through GSK3β activation. NDRG2 mediated the interaction between GSK3β and protein phosphatase 2A (PP2A), inducing the dephosphorylation of GSK3β at S9 by PP2A [70]. The interaction between NDRG2 and PP2A also activated PTEN, inhibiting AKT activation associated with cell survival and tumorigenesis [15,77]. Therefore, this shows that NDRG2 expression regulates pro/antiapoptotic protein levels, increasing the sensitivity of tumor cells to anticancer drugs (Figure 3). 

### 3.4. Metabolic Stress and NDRG2

**Figure 4:**
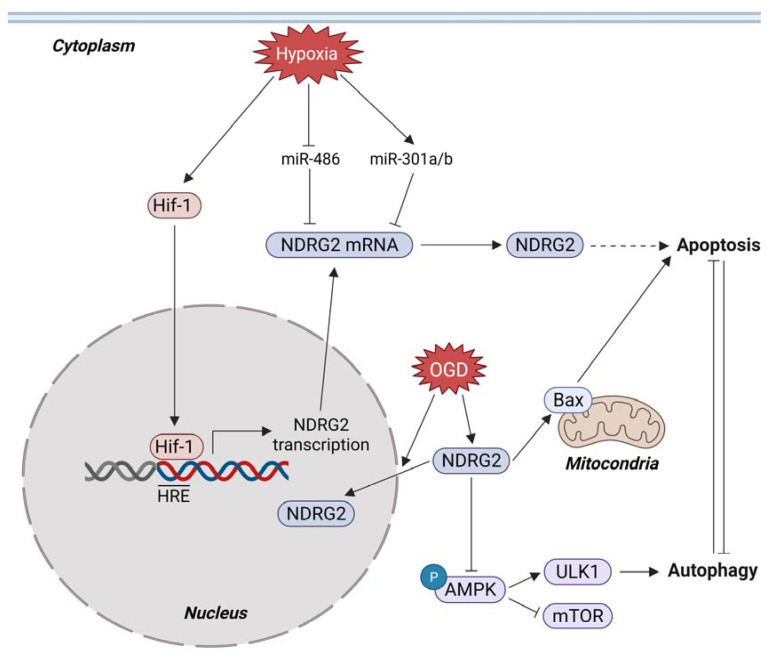
NDRG2 functions in the sensitivity of tumor cells to metabolic stresses (red), hypoxia, glucose deprivation, or both (OGD, oxygen-glucose deprivation). Hif-1, hypoxia inducible factor-1; AMPK, AMP-activated protein kinase; ULK1, Unc-51 like autophagy activating kinase 1; mTOR, mammalian target of rapamycin.

Oxygen is an essential factor that allows energy metabolism to perform biogenesis in cells, and hypoxia, the limitation of oxygen supply, is a crucial physiological stressor associated with various pathologies, such as stroke, infarction [78,79], brain injury [80], and tumorigenesis [81]. In tumor tissue, the rapid proliferation of tumor cells exceeds the vascular structures that surround the tumor and supply oxygen and nutrients to tumor cells. Hypoxia induces intratumoral oxygen gradients, contributing to tumor plasticity and promoting more aggressive and metastatic phenotypes of tumor cells [82,83]. Hypoxia-inducible factors (HIFs) are hypoxia-inducible transcription factors that contribute to the pathogenesis of pulmonary arterial hypertension, systemic hypertension, hereditary erythrocytosis, and cancer [84,85,86]. In a human lung cancer cell line, A594, mRNA and protein of NDRG2 were upregulated under hypoxic conditions [87]. HIF-1 directly bound to the putative hypoxia response element motif, from –188 to –183 bp, in the NDRG2 promoter. Silencing NDRG2 expression reduced apoptosis under hypoxic conditions, and miRNAs were shown to regulate NDRG2 expression under hypoxic conditions. In H9c2 cells modeling myocardial injury in vitro, hypoxia conditions inhibited miR-486 expression, which induced the upregulation of NDRG2 and increased apoptosis. NDRG2 is a target of miR-486, and silencing NDRG2 expression reduced the apoptosis of H9c2 cells under hypoxic conditions [88]. Additionally, other hypoxia response miRNAs, Mir-301a and miR-301b, were upregulated in LNCaP prostate cancer cells under hypoxic conditions, and decreased NDRG2 expression by directly targeting 3′ UTR of NDRG2. High levels of mir-301a and mir301b expression and silencing NDRG2 expression led to increased autophagy and reduced apoptosis [89,90]. Oxygen–glucose deprivation (OGD) can be used to study the effects of ischemia in cultured brain capillary endothelial cells. NDRG2 expression in C6 glioma cells was increased and translocated from cytosol to the nucleus, and increased the Bax/Bcl-2 ratio and apoptosis after OGD stress in a p53-dependent manner [91]. NDRG2 overexpression enhanced the proapoptotic effect and attenuated AMPK phosphorylation induced by glucose deprivation in a breast cancer cell, MDA-MB231 [92]. Under energy stress conditions, AMPK was shown to promote autophagy by reducing mTOR activation and inducing ULK1 activation through the phosphorylation of Ser 317 and Ser 777 [93,94,95]. There is a mutual inhibition relationship between autophagy and apoptosis; therefore, the inhibition of autophagy may induce apoptosis [96]. Although the NDRG2 function associated with autophagy has not been well studied, downregulated NDRG2 expression contributed to increased autophagy and reduced apoptosis in prostate cancer cells under hypoxic conditions [89,90]. Overall, NDRG2 contributes to the induction of apoptosis in various tissue cells under metabolic stress, such as oxygen deprivation or glucose deprivation (or both) (Figure 4).

## 4. Conclusions/Outlook

**Figure 5:**
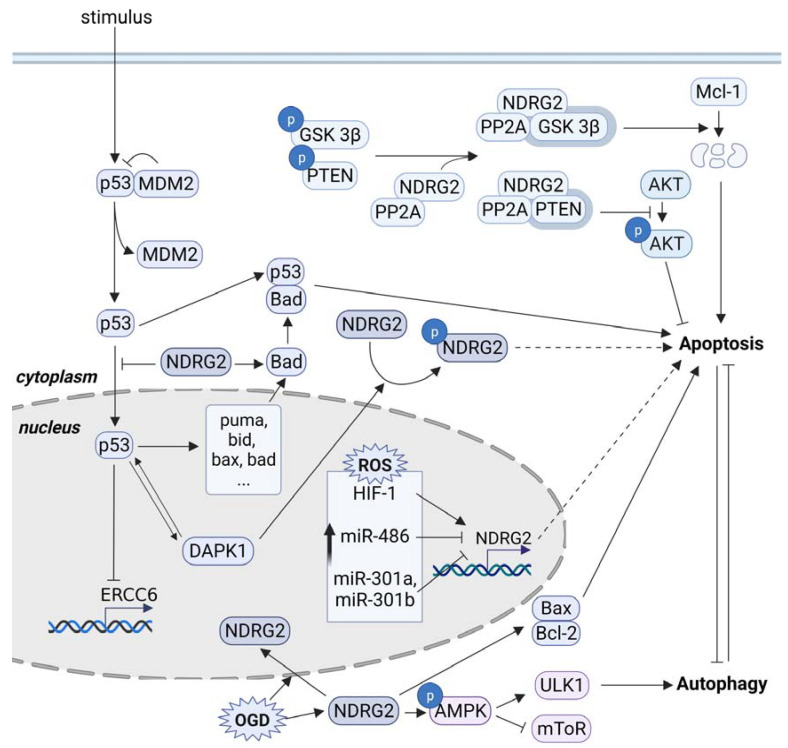
Overview of NDRG2 function in various stimuli-mediated apoptosis.

The expression of NDRG2 may be unique to different patients and is inversely associated with the clinical stage, disease-free survival rate, overall survival rate, or both rates. The expression of NDRG2 as a tumor-suppressive and stress-responsive gene could be negatively or positively regulated through the epigenetic regulation of the NDRG2 promoter, miRNA, c-Myc, p53, and HIF-1α under several physiological conditions. Overexpressed NDRG2 inhibits tumor cell proliferation and invasion/metastasis and sensitizes tumor cells to anticancer therapy by enhancing apoptosis (Figure 5). 

The resistance of tumor cells to apoptosis can promote aggressiveness and poor prognosis in various physiological conditions, such as hypoxia, nutrient deprivation, and anticancer drug treatment. Therefore, the identification of strategies to overcome the resistance of tumor cells to apoptosis will significantly help enhance the effectiveness of tumor treatment. Furthermore, NDRG2 expression is positively correlated with apoptosis induced by metabolic stressors, such as oxygen deprivation, glucose deprivation, or both, and anticancer drug treatments. Although mechanisms that regulate NDRG2 gene expression and NDRG2-mediated improvements of tumor cell apoptosis have been presented, the molecular mechanisms of these aspects are unclear. Although its functional domain is not well known, NDRG2 has recently been reported to interact with kinases or phosphatases (or both). Its potential as an adapter protein that mediates protein–protein interactions appears to induce antitumor phenotypes in several tumor cells. In the future, the continued discovery of and functional studies on proteins that interact with NDRG2 should be conducted. It is expected that tumor treatment strategies that account for the expression pattern of NDRG2 or that regulate NDRG2 expression should increase the efficiency of tumor treatments.
